# Reliability and validity of a questionnaire for self-assessment of complete dentures

**DOI:** 10.1186/1472-6831-14-45

**Published:** 2014-05-02

**Authors:** Yuriko Komagamine, Manabu Kanazawa, Yoshinori Kaiba, Yusuke Sato, Shunsuke Minakuchi

**Affiliations:** 1 Gerodontology and Oral Rehabilitation, Graduate School of Medical and Dental Sciences, Tokyo Medical and Dental University, Yushima, Bunkyo-ku, Tokyo 113-8549, Japan

**Keywords:** Complete denture, Questionnaire, Edentulous, Self-assessment, Reliability, Validity

## Abstract

**Background:**

Demand for complete denture treatment is expected to rise over several decades. However, to date, no questionnaire on complete dentures, as evaluated by edentulous patients, has been shown to be reliable and valid. This study sought to assess the reliability and validity of Patient’s Denture Assessment (PDA), which provides a multidimensional evaluation of dentures among edentulous patients.

**Methods:**

Patients, who had new complete dentures fabricated at the University Hospital of Dentistry, Tokyo Medical and Dental University through 2009 to 2010, were enrolled. The reliability of the PDA was determined by examining internal consistency and test-retest reliability. Internal consistency for all of the question items and the six subscales was measured using Cronbach’s α and average inter-item correlation coefficients among 93 participants. For 33 of these participants, test-retest reliability was determined at a 2 month-interval using the interclass correlation coefficients (ICCs) and 95% confidence interval for the summary scores and the six subscale scores. The PDA was validated in 93 participants by examining the difference in the summary score and the six subscale scores of the PDA before and after replacement with new dentures by the paired t-test. Ability to detect change was also tested in 93 patients using effect size.

**Results:**

The Cronbach’s α for the PDA ranged from 0.56 to 0.93. The average inter-item correlation coefficients ranged from 0.28 to 0.83. ICCs for the PDA ranged from 0.37 to 0.83. The paired t-test showed a significant difference between the summary score and the six subscale scores before and after replacement with new dentures (*p* < 0.05) and the effect size was 0.97.

**Conclusions:**

The PDA demonstrated good reliability by assessing internal consistency and test-retest reliability. In addition, the PDA demonstrated good validity by assessing discriminant validity. Thus, the PDA could help dentists obtain a detailed understanding of the patients’ perceptions in using their dentures.

## Background

In recent years, the National Epidemiological Survey data have revealed a decline in the proportion of edentulous people. However, given the simultaneous decline in the percentage of edentulous people and increase in the number of older people, the number of people requiring complete dentures in the United States is predicted to increase over the next 20 years [1]. Thus, the need for complete denture treatment is likely to remain substantial [2].

Patient-reported outcome measures, as recommended by the United States Food and Drug Administration, is a growing area within research [3], and the impact of treatment on patients has been attracting attention. This is also the case for prosthetic treatment: the success of complete denture treatment does not depend on the assessment by the dentist, but on patient self-assessment results. The patient self-assessment is an inexpensive and economical method with which to measure the success of the denture and can yield a large amount of data in a short period of time.

Patient satisfaction is rated using a questionnaire that is assessed via a Likert scale or the 100-mm visual analogue scale (VAS). Some patient satisfaction evaluation methods depend on only one question [4,5], and it is likely that such evaluations may not be reliable. For example, in dissatisfied patients, it is not known whether the dissatisfaction was caused by the lower or upper denture. In addition, patient satisfaction is usually not based on one factor, but on many factors, such as taste, ease of chewing, comfort, retention, pain, fit and aesthetics. Taking those factors into consideration, other evaluation methods have been introduced that comprise several questions to permit the multidimensional evaluation of patient satisfaction [6-9]. Limitations of these questionnaires are the lack of reliabilities and validities. On the other hand, in prosthetic treatment, one of the most widely used evaluation tools for measurements of oral health related quality of life (OHRQoL) is the Oral Health Impact Profile (OHIP). This instrument has been translated into many languages across the globe [10-16], which has allowed for comparisons of reliability and validity between OHIP in different regions. The OHIP assesses the impact of oral conditions on the quality of life using a frequency estimation of the disruption, such as dysfunction, discomfort and disability, in daily activities. Thus, negative-oriented measures composing the OHIP may be unable to capture positive changes in patients with no negative impacts recorded at baseline because the absence of a negative does not necessarily imply a positive [17].

Consequently, it is necessary to establish a multidimensional patient self-assessment specific to dentures that can capture both positive and negative denture-related effects and have reliability and validity. We first developed a questionnaire for the self-assessment of complete dentures [18] referred to as the Patient’s Denture Assessment (PDA). The PDA was developed from an original questionnaire that is a subjective assessment about oral, physical, and psychological conditions on daily functioning. In the PDA, some question items contain quality of life similar to the OHIP, but the PDA is absolutely assessment of dentures in themselves. Moreover, the PDA is the bipolar design, which could capture both negative and positive impacts on the patients. The conceptual of framework established for the PDA is in the following. The purposes of complete denture treatment are recoveries of three major functions; chewing, speech, and aesthetics. This three functions influence acceptances and expectations for dentures, which have impacts on patients’ perceptions, consciousness and feelings of dentures. The PDA assesses these impacts of complete denture treatment on the patients’ perceptions, consciousness and feelings of dentures. From the clinical point of view, the PDA will be used for the diagnosis, prognosis, and comparing the efficacy of prosthetic treatment. For example, if the association between the PDA scores before treatment and outcomes after treatment, such as numbers of denture adjustment, length of denture survival and denture survival rate is assessed, the difficulties of treatments and longevities of dentures as prognosis will be predictable from the PDA scores at pre-treatment diagnosis. However, at this point, reliability and validity of the PDA has not been confirmed yet. Thus, the purpose of this study is to demonstrate that the PDA was a reliable and valid instrument for application in clinical settings.

## Methods

### Participants

A total of 122 edentulous patients wearing upper and lower complete dentures and requiring new complete dentures were enrolled (55 men, 67 women; mean age, 74.4 years). All participants were recruited from undergraduate student treatment clinics at the University Hospital of Dentistry, Tokyo Medical and Dental University through 2009 to 2010. All participants could read and respond in Japanese. Of these 122 participants, 28 were excluded from the study as they did not complete the questionnaire, and one participant was unable to attend the study because of severe general condition. The remaining 93 participants were included in the analysis of internal consistency, discriminant validity and ability to detect change (Table 1). From the 93 participants, 33 participants were randomly selected to investigate test-retest reliability. The sample size and characteristics of the participants in each investigation are presented in Table 2. All participants gave their informed consent prior to enrolment. The study was approved by the institutional ethics committee of Tokyo Medical and Dental University (approval number 232). The patients were treated by undergraduate students under faculty staff supervision from the first visit. The technique for fabricating complete dentures involved primary and secondary impressions, recording of jaw relationships using occlusal rims, Gothic arch tracing, one or two trial insertions, and delivery of the new dentures. After delivery of the new complete dentures, adjustments to the dentures were made until a prosthodontist judged that further adjustment was unnecessary.

**Table 1:** Distribution of patients’ previous and current denture status according to upper and lower dentures (N = 93)

**Variable**	**Upper denture**	**Lower denture**
**Period of edentulousness**		
<1 year	11	21
1 year ≤ 3 years	2	4
3 years ≤ 5 years	12	6
5 years ≤ 10 years	13	12
≥10 years	55	50
Number of previous complete dentures		
0	3	11
1 – 3	74	65
4 – 6	12	13
≥7	4	4
Time since current complete dentures		
<0.5 years	28	27
0.5 years ≤ 10 years	33	35
≥10 years	32	31

**Table 2:** Sample size and characteristics of the participants for each investigation

**Type of investigation**	**N (male/female)**	**Mean age (years)**	**Year of enrolment**
**Internal consistency**	93 (44/49)	75.0	2009 & 2010
**Test-retest reliability**	33 (15/18)	74.5	2009
**Discriminant validity**	93 (44/49)	75.0	2009 & 2010
**Ability to detect change**	93 (44/49)	75.0	2009 & 2010

### Development of the patient denture assessment (PDA)

We previously developed and reported on our questionnaire for the self-assessment of dentures (Patient’s Denture Assessment or PDA) [18]. The PDA was developed from an original questionnaire comprising 39 question items that assessed factors related to dentures. A total of 39 question items covering six factors were obtained by the first factor analysis with Promax rotation. Sixteen question items met the exclusion criteria and were thus eliminated. Next, a second factor analysis with Promax rotation was performed. Finally, six factors were identified. Question items that were similar to other factors were eliminated and a few new question items were added. Throughout the process of developing the PDA, the questionnaire comprised 22 question items covering six factors, which were classified as ‘function’, ‘lower denture’, ‘upper denture’, ‘expectation’, ‘aesthetic and speech’ and ‘importance’. Table 3 represents 22 question items of the PDA, which were translated from Japanese to English for this article. It hasn’t been translated according to the guideline of translation. In the questionnaire, each item was measured using a 100-mm VAS, which consisted of a horizontal 100-mm line anchored by words representing the worst situation at the left extremity of the scale and words representing the best situation at the right extremity.

**Table 3:** Question items of the patient’s denture assessment (PDA)

**Subscale**	**Questionnaire items**
**Function**	Q1. How much pain do you feel?
	Q2. How easy is it for you to swallow food boluses and water?
	Q3. How well do you enjoy your meals?
	Q4*.* How worn out does your jaw feel?
**Aesthetics & speech**	Q5. How worried are you about other people watching?
	Q6. How easy is it for you to speak?
	Q7. How worried are you about your mouth?
	Q8. How often do your dentures click when chewing?
**Lower denture**	Q9. How often does food debris get stuck under your lower denture?
	Q10. How is your lower denture retained on the ridge?
	Q11. How does your lower denture fit?
	Q12. How uncomfortable is your lower denture?
**Expectation**	Q13. How satisfactory will the new dentures be?
	Q14. How problematic will the new dentures be?
	Q15. How well will the new dentures fit?
**Upper denture**	Q16. How often does food debris get stuck under your upper denture?
	Q17. How does your upper denture fit
	Q18. How often does your upper denture fall down?
**Importance**	Q19. How much do you consider your dentures as part of your body?
	Q20. How important are your dentures to you?
	Q21. How much can you care for your dentures without any difficulty?
	Q22. How at ease do you feel when wearing your dentures?

### Clinical measurements

All 93 participants were instructed to complete the PDA twice: before replacement (Before-1 PDA), and on completion of denture adjustment after replacement (After PDA). An additional PDA was performed before replacement (Before-2 PDA) for 33 participants randomly selected among the 93 participants. The interval between Before-1 PDA and Before-2 PDA was two months. Each subscale score was calculated by summing the values of the question items corresponding to each subscale. Moreover, the summary score of all the question items was calculated.

### Reliability and validity

Internal consistency of the PDA was assessed by Cronbach’s α and average inter-item correlation. Before-1 PDA scores from the 93 participants were used for an assessment of internal consistency. Cronbach’s α for summary score of 0.70-0.80 are considered satisfactory for a reliable comparison between groups and more than 0.90 are required for clinical usefulness of the instrument [19]. The test-retest reliability was assessed by determining the interclass correlation coefficients (ICCs) and 95% confidence interval of the test-retest difference for 33 participants. Factor analysis is reported in previous study [18]. To evaluate discriminant validity, we assessed the difference between Before-1 PDA and After PDA scores in 93 patients using a paired t-test. Values of *p* < 0.05 were considered significant for the paired t-test.

### Ability to detect change

Effect size was used to investigate the ability to detect change to the treatment. Ability to detect change to the questionnaire was assessed in 93 participants by calculating the effect size. Effect size is one of the most commonly used methods for interpreting a change in scores [20]. The effect size was evaluated by calculating the difference between the means of the summary scores of Before-1 PDA and After PDA and dividing the difference by the standard deviation (SD) of the summary scores of Before-1 PDA [21]. To facilitate decisions regarding the clinical importance of the observed change in the measure, some benchmarks have been proposed; a value of 0.2 or less, 0.5 and 0.8 or greater has been proposed to represent low, moderate and high ability to detect change, respectively [22].

### Statistical analysis

Internal consistency of the PDA was assessed by Cronbach’s α and average inter-item correlation. Test-retest reliability was assessed by the interclass correlation coefficients (ICCs) and 95% confidence interval. Discriminant validity was assessed by a paired t-test between PDA scores before and after treatment. Values of *p* < 0.05 were considered significant for the paired t-test. Ability to detect change was investigated by effect size. SPSS version 16.0 (SPSS Japan, Tokyo, Japan) was used for all analyses.

## Results

Table 4 provides the mean and SD of the Before-1 and After PDA scores. It’s notable that the some question items in the ‘importance’ subscale exhibited a ‘ceiling effect’. In addition, the ‘lower denture’ and ‘expectation’ subscales had lower means for the Before-1 PDA scores than that seen with the other subscales.

**Table 4:** Mean values and standard deviations (SDs) for before-1 and after patient’s denture assessment (PDA) scores (N = 93)

**Subscale**	**Items**	**Mean ± SD**	
		**Before-1**	**After**
**Function**	Q1	74 ± 30	83 ± 24
	Q2	78 ± 24	87 ± 18
	Q3	72 ± 28	83 ± 23
	Q4	74 ± 29	87 ± 21
**Aesthetics & speech**	Q5	67 ± 34	88 ± 20
	Q6	66 ± 33	85 ± 22
	Q7	58 ± 37	86 ± 21
	Q8	60 ± 37	72 ± 31
**Lower denture**	Q9	41 ± 35	70 ± 30
	Q10	45 ± 34	77 ± 28
	Q11	43 ± 35	82 ± 23
	Q12	47 ± 36	79 ± 26
**Expectation**	Q13	64 ± 38	87 ± 20
	Q14	51 ± 40	80 ± 28
	Q15	54 ± 42	82 ± 27
**Upper denture**	Q16	60 ± 36	83 ± 24
	Q17	71 ± 33	89 ± 18
	Q18	72 ± 33	91 ± 17
**Importance**	Q19	88 ± 23	90 ± 20
	Q20	95 ± 11	97 ± 6
	Q21	75 ± 26	84 ± 21
	Q22	79 ± 27	88 ± 18

Before-1, questionnaire before replacement; After, questionnaire after replacement.

### Reliability and validity

The Cronbach’s α score for all question items was 0.91 and fulfilled the criterion for clinical usefulness. For the six subscales, it ranged from 0.56 to 0.93. The average inter-item correlation for all question items was 0.59 and for the six subscales, it ranged from 0.28 to 0.83 (Table 5). The ICC of the summary score was 0.78 and the ICCs of the six subscales ranged from 0.37 to 0.83 (Table 6). The results of the assessment of discriminant validity are presented in Table 7. The paired t-test showed a significant difference between the Before-1 PDA and After PDA scores.

**Table 5:** Cronbach’s α and average inter-item correlation coefficients assessed by Before-1 PDA scores (N = 93)

**Scales**	**Cronbach’s α**	**Average inter-item correlation coefficient**
**Summary score**	0.91	0.59
**Function**	0.85	0.59
**Aesthetics & speech**	0.83	0.54
**Lower denture**	0.89	0.67
**Expectation**	0.93	0.83
**Upper denture**	0.86	0.67
**Importance**	0.56	0.28

Before-1, questionnaire before replacement; PDA, Patient’s Denture Assessment.

**Table 6:** Test-retest reliability assessed by before-1 and before-2 patient’s denture assessment (PDA) scores (N = 33)

**Scales**	**ICC**	**95% CI**
**Summary score**	0.78	[0.61, 0.89]
**Function**	0.74	[0.54, 0.86]
**Aesthetics & speech**	0.77	[0.69, 0.91]
**Lower denture**	0.83	[0.46, 0.83]
**Expectation**	0.37	[0.03, 0.63]
**Upper denture**	0.69	[0.59, 0.88]
**Importance**	0.59	[0.32, 0.77]

Before-1, questionnaire before replacement; Before-2, second questionnaire before replacement within 2 months of the first; After, questionnaire after replacement; ICC, interclass correlation coefficients CI, Confidential Interval.

**Table 7:** Results of the paired t-test for before-1 and after patient’s denture assessment (PDA) scores

**Scales**		**Mean ± SD**	***P***
**Summary score**	**Before-1**	1433 ± 430	< 0.00
	**After**	1850 ± 340	
**Function**	**Before-1**	298 ± 93	< 0.00
	**After**	340 ± 74	
**Aesthetics & speech**	**Before-1**	251 ± 116	< 0.00
	**After**	331 ± 73	
**Lower denture**	**Before-1**	176 ± 122	< 0.00
	**After**	307 ± 93	
**Expectation**	**Before-1**	169 ± 113	< 0.00
	**After**	249 ± 69	
**Upper denture**	**Before-1**	202 ± 90	< 0.00
	**After**	263 ± 43	
**Importance**	**Before-1**	336 ± 59	< 0.00
	**After**	360 ± 48	

Before-1, questionnaire before replacement; After, questionnaire after replacement.

### Ability to detect change

The mean summary scores changed from 1433 for the Before-1 PDA score to 1850 for the After PDA score. The SD of the summary scores of the Before-1 PDA was 430 and the effect size for the summary score was 0.97.

## Discussion

In the present study, both the reliability assessed by internal consistency and test-retest reliability and the validity assessed by discriminant validity of the PDA were satisfactory. Therefore, the PDA is suitable for use in clinical settings.

The internal consistency of the PDA was assessed using two measures: average inter-item correlation and Cronbach’s α. Cronbach’s α is computed from the SDs of the question items and the SD of the total scores. As the difference between the SDs for question items of a subscale becomes larger, the Cronbach’s α of the subscale decreases [23]. In the PDA, each subscale comprised three or four question items, which often resulted in a smaller value of Cronbach’s α in each subscale. Nevertheless, five out of the six subscales presented with a value of 0.8-0.9. Only the ‘importance’ subscale presented with a low Cronbach’s α of 0.56.

It is important to note that, in this study, in describing the importance of dentures, Q20 exhibited a ‘ceiling effect’. This was because all patients had used complete dentures previously and required new dentures, thus complete dentures were important for them. For this reason, the SDs of Q20 were small for most of the patients as compared with that in the other three question items for this subscale. Moreover, the difference between the Before-1 PDA and After PDA scores mean values of Q21 or Q22 was large with wide variance. On the other hand, the difference of Q19 or Q20 was small with narrow variance. The question items of Q21 and Q22 were added after second factor analysis. Thus, the directions of the construct of Q19 or Q20 might be different from Q21and Q22. These led to a small Cronbach’s α, the average inter-item correlation and the impact in the ‘importance’ subscale. By comparison, the highest average inter-item correlation coefficient was in the ‘expectation’ subscale. It is likely that Q13 and Q15 (Table 3) deliver similar content, which may account for the highest score of the average inter-item correlation coefficients in the ‘expectation’ subscale.

The ICCs of the test-retest reliability for the ‘expectation’ and ‘importance’ subscales were low as compared with other studies [10,11]. The reason for this discrepancy may be that the interval between the test-retest in our study was 2 months while other studies used a 1- or 2-week-interval. The ‘expectation’ subscale consisted of question items related to expectancy of improvement with new dentures and the ‘importance’ subscale consisted of question items related to the importance of dentures to patients, depending on the patients’ perception. The 2-month interval was sufficient time to enhance the relationship and trust between the patients and their operators, which may have influenced their expectation perceptions to new dentures and the importance of wearing dentures.

For the assessment of validity, there was a significant difference in the summary score and the six subscale scores before and after replacement of new dentures. Thus, it is suggested that the PDA can detect differences in patients’ self-assessment between previous and new dentures.

The ‘lower denture’ and ‘expectation’ subscales had lower means for the Before-1 PDA scores than that seen with the other subscales. This suggests that most of the participants selected for the study wore insufficient lower dentures and they had low expectations for the efficacy of their new dentures. Table 1 shows larger number of patients whose period of lower edentulousness was less than one year than upper edentulousness. Moreover, the previous study has shown that patients have a lower expectation for their new dentures when their rating for their previous dentures is low [24]. Therefore, it is suggested that shorter period of edentulousness and insufficient dentures might cause problems with lower dentures and following low expectations in the study.

In the present study, the effect size was 0.97. According to the already-described statement, it is suggested that 0.97 of the ability to detect change in the study represents high ability to detect change. The PDA after treatment was requested after the dentures had been completely adjusted, with all participants given adequate time for these adjustments. Thus, the negative effects of the previous dentures were no longer present and positive effects of the new complete dentures might explain the large effect size reported in the study.

This study demonstrated good reliability and validity of the PDA. Thus, this questionnaire could help dentists obtain a detailed understanding of the patients’ perceptions in using their dentures, which is considered valuable information for dentists when designing new dentures. In our previous study, we demonstrated that sufficient retention of lower dentures and appropriate appearance would lead to improved OHRQoL in edentulous patients who had previously been wearing insufficient lower dentures and/or dentures with an unsatisfactory appearance [18]. The PDA can be thus applied in the clinical situation. However, dentists should carefully interpret the results specific to the ‘importance’ subscale because of the ‘ceiling effect’ and the possibility of the difference direction between Q19 or Q20 and Q21 or Q22 seen in this study.

All participants in the study were recruited in a university hospital setting. Therefore, the reliability and validity of our test must be extrapolated to the general population besides patients in university hospitals. In addition, the questionnaire is presently written in Japanese only. It is recommended that the questionnaire is translated into other languages and evaluated for its reliability and validity with a view for its wider implementation in future studies.

## Conclusions

When used among edentulous patients requiring new complete dentures, the PDA demonstrated good reliability by assessing internal consistency and test-retest reliability. In addition, the PDA demonstrated good validity by assessing discriminant validity.

## Abbreviations

FDA: United States food and drug administration; OHRoL: Oral health-related quality of life; VAS: Visual analogue scale; OHIP: Oral health impact profile; PDA: Patient’s denture assessment.

## Competing interests

The authors declare that they have no competing interests.

## Authors’ contributions

YK contributed to the design of the study, data analysis and drafted the manuscript. MK, YK, YS and SM contributed to the design of the study, data collection and performed data analysis. All authors read and approved the final manuscript.

## Pre-publication history

The pre-publication history for this paper can be accessed here:

http://www.biomedcentral.com/1472-6831/14/45/prepub
